# Enhanced Resting-State Functional Connectivity With Decreased Amplitude of Low-Frequency Fluctuations of the Salience Network in Mindfulness Novices

**DOI:** 10.3389/fnhum.2022.838123

**Published:** 2022-03-03

**Authors:** Quan Gan, Ning Ding, Guoli Bi, Ruixiang Liu, Xingrong Zhao, Jingmei Zhong, Shaoyuan Wu, Yong Zeng, Liqian Cui, Kunhua Wu, Yunfa Fu, Zhuangfei Chen

**Affiliations:** ^1^Medical Faculty, Kunming University of Science and Technology, Kunming, China; ^2^Department of Magnetic Resonance Imaging, The First People’s Hospital of Yunnan Province, Kunming, China; ^3^Department of Clinical Psychology, The Second People’s Hospital of Yunnan Province, Kunming, China; ^4^Department of Psychiatry, The First Affiliated Hospital of Kunming Medical University, Kunming, China; ^5^Department of Clinical Psychology, The First People’s Hospital of Yunnan Province, Kunming, China; ^6^Department of Clinical Psychology, The Sixth Affiliated Hospital of Kunming Medical University, Yuxi, China; ^7^Department of Neurology, The First Affiliated Hospital, Sun Yat-sen University, Guangzhou, China; ^8^School of Information Engineering and Automation, Kunming University of Science and Technology, Kunming, China

**Keywords:** emotion regulation, functional connectivity (FC), mindfulness, amplitude of low-frequency fluctuation (ALFF), functional magnetic resonance imaging (fMRI)

## Abstract

Mindfulness and accordant interventions are often used as complementary treatments to psychological or psychosomatic problems. This has also been gradually integrated into daily lives for the promotion of psychological well-being in non-clinical populations. The experience of mindful acceptance in a non-judgmental way brought about the state, which was less interfered by a negative effect. Mindfulness practice often begins with focused attention (FA) meditation restricted to an inner experience. We postulate that the brain areas related to an interoceptive function would demonstrate an intrinsic functional change after mindfulness training for the mindful novices along with paying more attention to internal processes. To further explore the influence of mindfulness on the organization of the brain regions, both functional connectivity (FC) in the voxel and the region of interest (ROI) level were calculated. In the current study, 32 healthy volunteers, without any meditation experiences, were enrolled and randomly assigned to a mindfulness-based stress reduction group (MBSR) or control group (CON). Participants in the MBSR group completed 8 weeks of mindfulness-based stress reduction (MBSR) and rated their mindfulness skills before and after MBSR. All subjects were evaluated *via* resting-state functional MRI (rs-fMRI) in both baselines and after 8 weeks. They also completed a self-report measure of their state and trait anxiety as well as a positive and negative affect. Pre- and post-MBSR assessments revealed a decreased amplitude of low-frequency fluctuations (ALFF) in the right anterior cingulate gyrus (ACC.R), left anterior and posterior insula (aIC.L, pIC.L), as well as left superior medial frontal gyrus (SFGmed.L) in MBSR practitioners. Strengthened FC between right anterior cingulate cortex (ACC.R) and aIC.R was observed. The mean ALFF values of those regions were inversely and positively linked to newly acquired mindful abilities. Along with a decreased negative affect score, our results suggest that the brain regions related to attention and interoceptive function were involved at the beginning of mindfulness. This study provides new clues in elucidating the time of evaluating the brain mechanisms of mindfulness novices.

## Introduction

By cultivating a state of sustained attention to internal processes in the present moment with acceptance and without judgment (Kabat-Zinn, 1990), mindfulness and accordant interventions have shown benefits with various psychological or psychosomatic problems when they are used as complementary treatments (Goyal et al., 2014). Mindfulness has been proven to be effective in alleviating chronic pain (Priddy et al., 2018), relieving the symptoms of depression (Marchand, 2012), anxiety (MacDonald and Olsen, 2020), or even reducing substance cravings of addiction (Enkema and Bowen, 2016). Moreover, mindfulness becomes popularized in recent years as an aid to help with the promotion of psychological well-being in non-clinical populations (Kingston et al., 2007). Mindfulness meditators often report less acceptance (Brown and Ryan, 2003), more acceptance, and a better regulation of negative affect (Wenzel et al., 2020). For these reasons, mindful practices have been incorporated into daily lives rather than as independent psychological interventions. The benefits of mindfulness have often been considered in their association with emotion regulation (Guendelman et al., 2017). This study convinces us that both attention and attitude were involved in the contribution of mindfulness to emotion regulation (Cavicchioli et al., 2018). By reappraising and focusing on the present moment, individuals become aware of their momentary sensations in the background of broadened attention and learn to adapt into new situations. Moreover, the experience of accepting feelings that arise, in a non-judgmental way, makes it easier to adapt to undesirable situations (Murphy and Lahtinen, 2015). The state of well-being could be achieved simply by accepting instead of fighting to control emotions. Corresponding to this notion, a recent study showed a significant association between mindfulness ability (especially non-judgmental acceptance facet) and the lesser use of emotion regulation strategies. It also proved and indicated that mindfulness may facilitate better well-being by lessening the need for strenuous emotion regulation.

Accordingly, mindfulness practice can generally be divided into two categories, i.e., focused attention (FA) and open monitoring (OM) (Lutz et al., 2008; Britton et al., 2018). FA practice is normally considered as an attentional skill in which selective attention of a chosen object, such as the sensation of breathing. Nevertheless, OM emphasizes de-selection, non-reactive monitoring of internal and external sensation, and entails non-judgmental awareness of experience. Basically, the attention regulation is the common link for both kinds of methods, which are comprehensively combined in traditional practice. In mindfulness practice, FA and OM are used jointly in enhancing the process of detecting mind wandering, configuring attention resources, and reducing habitual behavior. Normally, FA improves attentional resources in the first place, which in turn may reduce emotional interference (Farb et al., 2012). The process could be summarized as an interaction between attention regulation (Hasenkamp and Barsalou, 2012) and rising awareness (Kwee, 1995; Raffone et al., 2010; Keng et al., 2011; Vago and Silbersweig, 2012). A non-judgmental would lead to a certain perspective change, nominated as “decentering” (Fresco et al., 2007). With repeat practice, automatic attentional habits were gradually noticed. Concomitantly, metacognitive awareness was cultivated, which led to a reasonable distribution of attentional resources. Ultimately, concurrent monitoring of multiple present-moment experiences took place (Carmody, 2009). Detachment, decentering, or deautomatization was entailed by the previously mentioned procedure and rumination was reduced (Gu et al., 2015), increased levels of mindful attention and awareness were finally achieved (Shapiro et al., 2008). The underlying brain function may shed light on the mechanisms of mindfulness (Brown et al., 2007; Duquette, 2017).

Meanwhile, an accumulating body of evidence in neuroimaging is revealing the effects of mindfulness on the structure of the brain (Grant et al., 2010; Lazar et al., 2011; Kurth et al., 2014; Lu et al., 2014), activation (Farb et al., 2007; Braden et al., 2016; Tomasino and Fabbro, 2016), or neural connectivity (Doll et al., 2015; Murakami et al., 2015; Taren et al., 2015; Anthony et al., 2016) after 8 weeks of mindful induction (Gotink et al., 2016). Attention (Tsai and Chou, 2016) networks were particularly highlighted (Dickenson et al., 2013; Peters et al., 2016; Tomasino and Fabbro, 2016). Distinctive parts of the brain were regulated by the three subfunctions of attention (Tsai and Chou, 2016). While the involvement of subnetworks is sequential according to how much effort is needed for the maintenance of the different states (Tang et al., 2012). Recent studies have also revealed intrinsic functional connectivity (FC). Inter- and/or intra-attention networks were modified with mindfulness (Doll et al., 2015; Roland et al., 2015). To be specific, much effort was required to achieve the meditative state at the beginning. The lateral prefrontal cortex (PFC) and parietal cortex are mostly involved in voluntary control (Farb et al., 2007; Tang et al., 2009, 2012), whereas with the anterior cingulate cortex (ACC) less effort is invested (Tang et al., 2009, 2012; Hölzel et al., 2011; Tang, 2011). In the middle stage of meditation, practitioners began to notice distractions of the wandering mind that was decreased as awareness increased. With an appropriate effort, the participants’ meditation skills and attention control increased. Hence, the brain regions related to awareness and attention switched, namely the salience network (SN), dorsolateral prefrontal cortex (dlPFC), and posterior parietal lobule could be activated (Hasenkamp and Barsalou, 2012; Malinowski, 2012; Tang et al., 2012). Along with the practice, one became proficient in the awareness of distraction and the switching of attention. Theoretically, the maintenance of a mindful state may be achieved with little or no effort in the advanced stage. One is mindful of the present moment, whether it includes a particular object or all salient stimuli. Thus, attention is focused and broadened and “conflict monitoring is used to a lesser degree” (Barinaga, 2003). With the decrease of attention control, the obtaining and maintenance of mindfulness state became much fluent. The state may be supported by the ACC, left insula, and striatum (Tang et al., 2012). Besides, the midline structure was functionally associated “getting caught up in” experience, the deeper one was immersed the stronger the activation it became (Northoff et al., 2006). Studies of experienced meditators that the indicated posterior cingulate gyrus (PCC) was activated in “distraction” and deactivated in “concentration” (Brewer et al., 2013). Synchrony or connectivity between frontal and parietal lobes may also reflect the conscious awareness of the present moment (Taylor et al., 2013). Therefore, a certain neuronal basis was detected to underpin the brain functional change following the “react” to “respond” (Doran, 2014) transition.

Generally, mindfulness often begins with FA meditation (Hasenkamp and Barsalou, 2012), which is restricted to a specific object (normally the sensory experience of the breath). The calmness state was not so hard to achieve in mindfulness beginners. It would be of great value to evaluate the underlying mechanism that may provide new clues in relation to the psychosomatic intervention. Therefore, we postulate that the brain areas responsible for an interoceptive function would present with an intrinsic functional change after mindfulness training in novices, following the procedure of learning to pay more attention to internal processes (especially the body sensation), the brain areas responsible for an interoceptive function would present with an intrinsic functional change after mindfulness training. To test the prediction, resting-state functional MRI (rs-fMRI) was used to assess brain activation in mindfulness meditation beginners before and after 8-week of mindfulness practice as well as in matched controls. As a reliable index for the evaluation of neuronal activation, the regional amplitude of low-frequency fluctuations (ALFF) in the conventional frequency band (0.01–0.08 Hz) was adopted (Zang et al., 2007). It has been investigated in numerous neuropathological and physiological states since its proposition (Zang et al., 2007; Han et al., 2011; Wang et al., 2011; Liu et al., 2012; Pan et al., 2014; Xiao et al., 2015). Considering the current topic, it has been proven to be effective on analyzing the neural basis of both FA and mindfulness meditation (Miyoshi et al., 2019; Yang et al., 2019). In addition, to further explore the influence of mindfulness on the organization of the brain regions, FC was calculated in both voxel and the region of interest (ROI) level.

## Materials and Methods

### Participants

In total, 32 healthy volunteers (16 men) without any mindfulness or meditation experiences were recruited in this study. They were randomly assigned to the mindfulness-based stress reduction group (MBSR) or control group (CON) (16:16). Those subjects in the MBSR group periodically attended MBSR practice for 8 weeks. While participants in the CON group merely accomplished relax practice during the same period.

All subjects were recruited through poster advertisements from the local community. They were interviewed by the two experienced psychiatrists using the SCID-I/NP (non-patient version) to exclude subjects with any history of neuropsychiatric illness. All participants were Han Chinese, right-handed, and assessed using the Annett Handedness Scale (Annett, 1970). For both groups, subjects with organic brain disorders, a history of alcohol or drug abuse, pregnancy, or severe physical illnesses were excluded. All participants provided written informed consent. The study was approved by the Ethical Committee of Kunming University of Science and Technology (ethical approval number: 2013JC003).

### Mindfulness Meditation Training

Following the traditional setting (Kabatzinn, 2010), the 8-week MBSR program consisted of weekly group meetings and homework. Each weekly meeting lasted for 2 h each time, and was divided into 3 parts, a theoretical part (30 min), a practical part (60 min), and a debriefing part. In the theoretical part, MBSR and underlying neuroscience were introduced to participants. In the following practice part, sitting meditation exercises were conducted *via* simple physical and breathing exercises, focusing attention on thoughts and feelings that came in without dwelling on any of them. All classes were taught by a senior teacher having experience in mindfulness teaching over 5 years. The daily homework practice was composed of formal and informal practices. The former lasted for 30 min each day, took the form of a body scan, sitting meditation, floor yoga, the mountain/lake meditation, or the loving kindness meditation. Each week, participants were asked to fill a formal practice sheet that was tailored for that week and guided that week’s practice. Aiming to integrate the learnings and practices into daily life, the informal practice was mainly simple awareness, i.e., bringing mindful awareness to routine activity, pleasant/unpleasant events, or communication situations. Additional techniques, such as 1-min breathing space (Ward, 2014) and recognize, accept, investigate, non-identification (RAIN) (Brewer et al., 2011) process were involved in informal practice help to notice automatic reaction and open awareness. At the end of each day, the participants took just 5 min or so to reflect on the day, using that week’s informal practice sheet as a guide.

### Process Measures

Mindfulness skills were assessed with the 39-item Chinese version of the Five Facet Mindfulness Questionnaire (FFMQ-C) (Hou et al., 2014). FFMQ, which is sensitive to a change in mindfulness-based interventions (Gu et al., 2015), consists of five subscales: observing, describing, acting with awareness, non-judging of inner experience, and non-reactivity to inner experience. In addition, to measure state and trait anxiety, the Chinese version of the State-Trait Anxiety Inventory (STAI) (Shek, 1988) was adopted. The STAI is a 40-item self-report questionnaire used to measure both current anxiety and state (20 items) anxiety. The Positive and Negative Affect Schedule (PANAS) was also applied both before and after the MBSR training to explore emotional states for all subjects. The PANAS is a 20-item self-report scale that measures positive and negative mood states in relation to the time frame of the previous week. Both negative and positive affect scales consist of 10 adjectives describing corresponding emotions, respectively. Participants rate the degree to which they feel each emotion on a scale from 1 (very slightly or not at all) to 5 (extremely).

### MRI Data Acquisition

Functional MRI data were acquired using a Signa Excite 3.0 Tesla scanner (GE Healthcare, Waukesha, WI, United States) at the First People’s Hospital of Yunnan Province with a spin echo-planar imaging (EPI) sequenced with an eight-channel phase array head coil. Data sets were aligned to the anterior–posterior commissure (AC-PC) line, using the following scan parameters: repetition time = 3,000 ms; echo time = 40 ms; image matrix = 64 × 64; field of view = 24 cm × 24 cm; 34 contiguous slices of 4 mm and without a gap; and restraining foam pads were used to minimize head motion. Subjects were instructed to simply relax, to keep their eyes closed, and to remain awake and perform no specific cognitive exercise.

### fMRI Image Processing

Resting-state fMRI data preprocessing was carried out by using Data Processing Assistant for Resting-State fMRI (DPARSF, V2.2^1^), which was based on SPM8^2^ and the Resting-State fMRI Data Analysis Toolkit plus (RESTplus, V1.1, see Text Footnote 1). The first 10 volumes were discarded to allow for steady-state magnetization. Further data preprocessing included slice timing correction, head motion correction, spatial normalization, and smoothing. Spatial normalization was performed by using the standard EPI template from the Montreal Neurological Institute (MNI). Then, linear detrending and temporal bandpass (0.01–0.08 Hz) filtering were performed to remove low-frequency drifts and physiological high-frequency noise. In addition, the effects of the global mean signal, white matter, and cerebrospinal fluid (CSF) were regressed out by using the default masks included in the package.

### Amplitude of Low-Frequency Fluctuations Calculation

Amplitude of low-frequency fluctuations was calculated using the RESTplus software. Spatially normalized data were smoothed with a 6 mm full width at half maximum (FWHM) Gaussian kernel prior to ALFF calculation. The time series was first converted to the frequency domain using a Fast Fourier Transform for a given voxel. The square root of the power spectrum was computed and then averaged across the predefined frequency interval. This averaged square root was termed as the ALFF at the given voxel (Zang et al., 2007). ALFF measures the absolute strength or intensity of spontaneous low-frequency oscillations (typically 0.01–0.08 Hz). Under the studied frequency ranges, ALFF at each voxel was computed for each subject, and it was further applied with Fisher’s *r*-to-*z* transformation to obtain a comparable *z*-value instead of the original.

### Statistical Analyses

The independent sample *t*-tests and the chi-squared test were used to compare demographic data between MBSR and CON groups with SPSS 13.0 software (SPSS, Chicago, IL, United States).

### Voxel-Wise Comparison of Amplitude of Low-Frequency Fluctuations Maps

Voxel-based comparisons of ALFF maps were performed to detect the intergroup and intragroup differences. The preprocessed data were analyzed as the two-sample/paired *t*-test by fitting the general linear model (GLM) in SPM8. The results at *p* < 0.05 at the voxel level, and *p* < 0.05 at the cluster level, with family-wise error (FWE) correction and cluster > 50 voxels for ALFF were considered to be statistically significant.

### Network Analyses

Two levels of FC were conducted. Firstly, five spherical regions of interest (ROIs) were defined in the regions of ALFF alteration detected in the current study. The ROIs were defined as spheres of 6 mm radius centered on peak coordinates of regional differences in ALFF maps among practitioners. In the voxel-wise seed-based FC analysis, a whole-brain FC map for each seed was generated. In the ROI-wise analysis, a 5 × 5 correlation matrix was created for each subject. For the correlation coefficients, Fisher’s *r*-to-*z* transformation was applied to obtain a comparable *z*-value instead of the original *r*, and then a difference of any paired *z*-value was calculated by using a paired *t*-test. For the voxel level analysis, *p* < 0.05 with FWE correction and cluster > 50 voxels were considered as the networks linked to the seeds. Bonferroni correction was applied in the ROI level FC analysis.

### Correlation Analysis

Pearson correlation analysis was used for assessing associations between the coupling of ROIs (mean ALFF) and mindfulness (FFMQ) scores. The analyses were conducted using SPSS.

## Results

### Demographic Description

In total, 16 subjects and demographically matched controls were recruited. There were no significant differences in demographic variables (*p* > 0.05) between the groups (Table 1).

**TABLE 1:** Demographic data for all subjects.

	MBSR	CON
Age (years)	27.63 (1.25)	28.06 (1.60)
Gender (male: female)	8:8	8:8
Years of education	17.00 (0.57)	15.17 (0.67)

*MBSR, mindfulness-based stress reduction training group; CON, control group; values are given as a number or mean (SE). No statistically significant differences between the groups (p > 0.05).*

### Change in Mindfulness Ability and Affective States

A paired *t*-test was conducted to analyze mindfulness ability and affective state change in the MBSR group. The total score and subscale scores except for the non-judgment of FFMQ indicated a significant raise of self-report mindfulness after MBSR training (*p* < 0.05). Both state and trait anxiety scores showed no difference between baseline and post-training evaluation (*p* > 0.05). The negative effect score of PANAS also significantly decreased after training (*p* < 0.01) (Table 2).

**TABLE 2:** The Five Facet Mindfulness Questionnaire (FFMQ), the State-Trait Anxiety Inventory (STAI), and the Positive and Negative Affect Schedule (PANAS) scores before and after meditation training.

	Before training	After training	*T*-value
**Mindfulness score (FFMQ)**			
Observe	23.06 (1.54)	27.00 (1.27)	**−3.134**
Describe	22.37 (1.09)	28.44 (1.19)	**−6.235**
Awareness	20.31 (1.15)	27.81 (1.11)	**−3.890**
Non-judgment	23.63 (1.32)	26.63 (1.22)	**−**1.324
Non-reactivity	19.50 (1.09)	22.31 (1.31)	**−2.397**
Total	108.876 (4.21)	132.19 (3.84)	**−5.307**
**Anxiety state (STAI)**			
State Anxiety Inventory	45.56 (1.49)	41.87 (1.15)	2.112
Trait Anxiety Inventory	44.43 (1.57)	44.12 (1.43)	0.168
**PANAS**			
Positive affect	30.84 (0.86)	30.38 (0.95)	1.225
Negative affect	16.63 (0.89)	12.88 (0.62)	**5.616**

*Values are given as group means (SE). Bold values indicate p < 0.05.*

### General Linear Modeling Results

**FIGURE 1:**
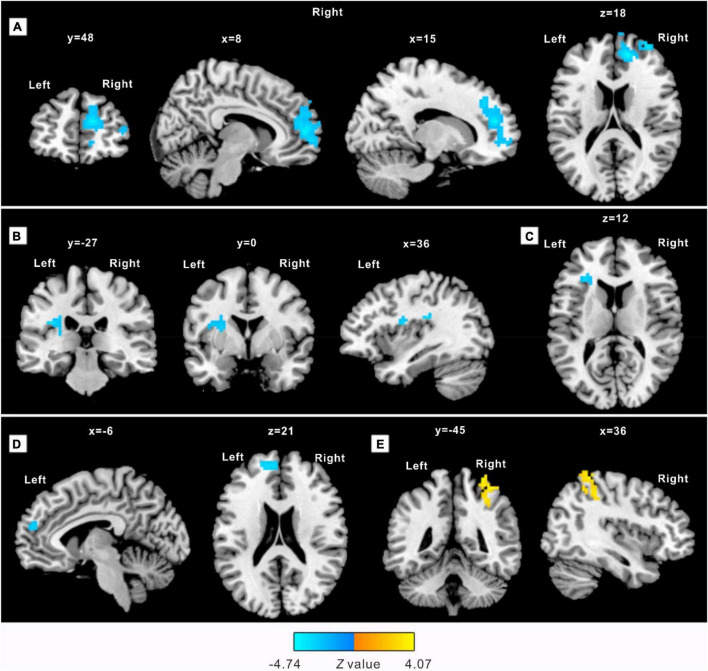
Practitioners demonstrate a significantly different amplitude of low-frequency fluctuations (ALFF) after an 8-week practice. The clusters detected with decreased ALFF include: **(A)** right anterior cingulate gyrus (ACC.R), extending into the right middle frontal gyrus; **(B)** left posterior insula (pIC.L), extending into the left putamen; **(C)** left anterior insula (aIC.L), extending ventrolateral into the left middle frontal gyrus; **(D)** peak in the left superior medial frontal gyrus (SFGmed.L), extending into the left superior dorsolateral frontal gyrus. A cluster located in the right postcentral gyrus (PostCG.R) extending into the right superior parietal gyrus **(E)** showed increased ALFF. Sections are shown in sagittal, axial, and coronal planes with Montreal Neurological Institute (MNI) coordinates of the selected sections representing the peak in the *x*-, *y*-, and *z-* direction.

An intergroup whole-brain contrast analysis between the MBSR and CON group in baseline was performed. Then, intragroup analyses were conducted between the baseline and 8-week assessments in both the groups separately. No significant difference was detected in the first contrast, neither in the intragroup comparison in the CON group (*p* > 0.05). In the contrast between baseline and post-training assessment in the MBSR group, decreased ALFF in the right anterior cingulate gyrus (ACC.R), left anterior and posterior insula (aIC.L, pIC.L), as well as the left superior medial frontal gyrus (SFGmed.L) was detected in case of practitioners. Increased ALFF was observed in the right postcentral gyrus (PostCG.R) (Table 3 and Figure 1).

**TABLE 3:** Brain areas with a change of amplitude of low-frequency fluctuations (ALFF) after MBSR training.

Region	BA	*t*-value	*P*-value	Cluster size	Peak MNI-coordinates		
					*x*	*y*	*z*
ACC.R	32	7.43	≤0.001	509	15	48	18
pIC.L	48	6.26	0.0089	119	−36	−27	24
SFGmed.L	10	5.32	0.0464	83	−6	54	21
aIC.L	48	5.25	0.0063	127	−30	27	12
PostCG.R	7	−5.65	0.0005	190	30	−45	72

Shown are results of paired t-tests of regional ALFFs in MBSR group after 8 weeks’ training compared with baseline [cluster level, family-wise error (FWE) corrected, p < 0.05]. ACC.R, right anterior cingulate cortex; pIC.L, left posterior insula; SFGmed.L, left medial superior frontal gyrus; aIC.L, left anterior insula; PostCG.R, right postcentral gyrus; BA, Brodmann area; MNI, Montreal Neurological Institute.

### Pre- and Post- mindfulness-Based Stress Reduction Group Comparison of Functional Connectivity

**FIGURE 2:**
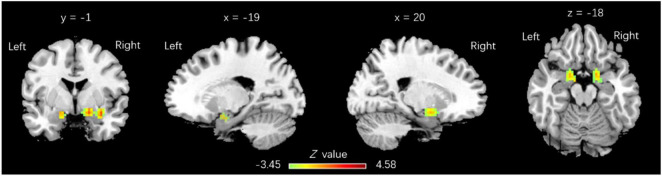
Regions with functional connectivity (FC) increase linked to pIC.L. pIC.L-related FC with a significant increase was observed in bilateral amygdala, with 90 voxels on the **left** (*x* = –18, *y* = 3, *z* = –12; *p* = 0.016) and 185 voxels on the **right** (*x* = 18, *y* = 3, *z* = –15; *p* = 0.017), family wise error (FWE) corrected.

As elaborated in the section “Materials and Methods,” five spherical ROIs were chosen in the regions with ALFF alteration detected in the current study. Based on all 5 seed ROIs, voxel-wise functional analysis revealed strengthened FC between pIC.L and bilateral amygdala [cluster size: 90 voxels on the left (peak MNI coordinates *x* = −18, *y* = 3, *z* = −12; *p* = 0.016) and 185 on the right (MNI coordinates *x* = 18, *y* = 3, *z* = −15; *p* = 0.017), FWE corrected] (Figure 2). In the ROI-wise analysis, the dominance of coupling between the right ACC and left medial superior frontal gyrus (SFGmed) attenuated [*r*_1_ = 0.60 ± 0.25, *r*_2_ = 0.33 ± 0.22, *t*_(15)_ = −3.215, *p*_corr_ = 0.003], while the dominance of coupling between ACC.R and aIC.R enhanced after MBSR training (*r*_1_ = −0.03 ± 0.16, *r*_2_ = 0.15 ± 0.27, *t* = −3.221, *p*_corr_ = 0.022).

### Association Between Coupling of Region of Interests and Mindfulness Scores

**FIGURE 3:**
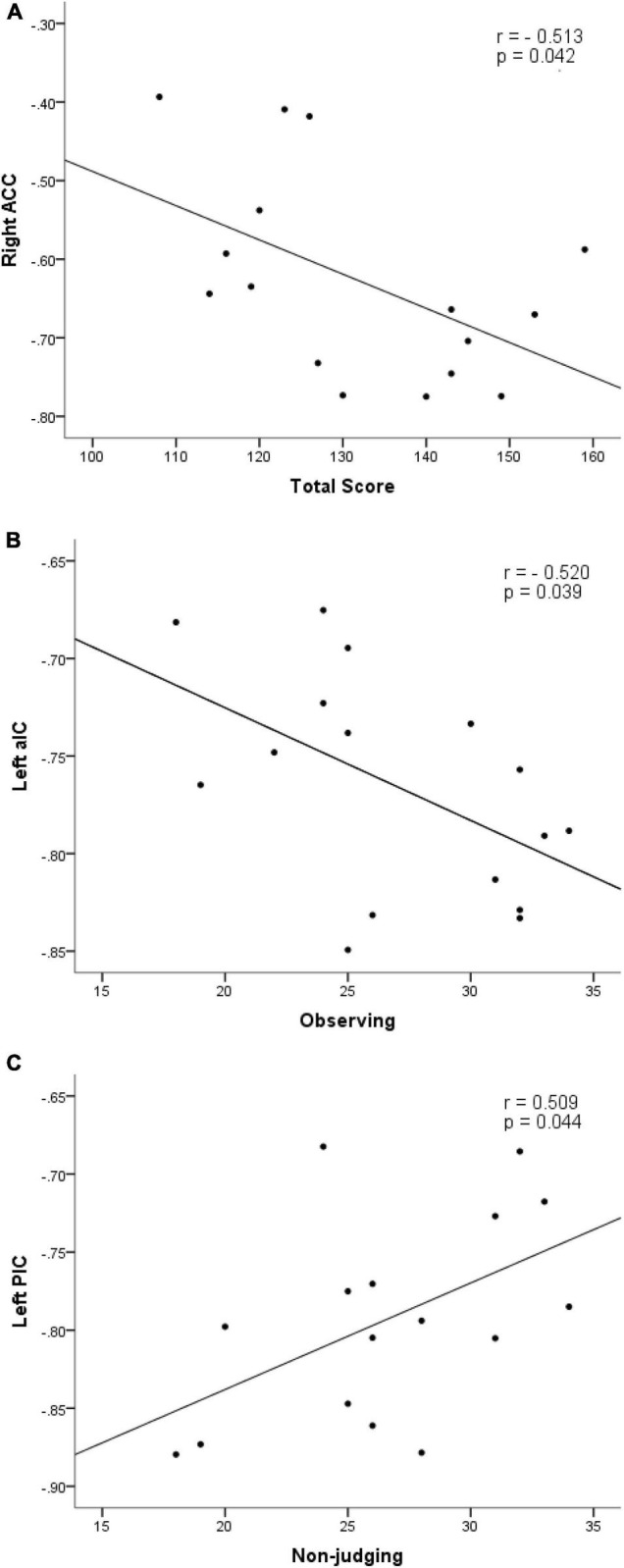
Scatter plot diagrams. The plots and fitted lines indicate: inverse correlations between the ALFF values of ROIs in right anterior cingulate cortex and aIC.L cortex with the total score (*r* = –0.513, *p* = 0.042) **(A)** and observing score (*r* = –0.520, *p* = 0.0392) **(B)**; a positive correlation between the ALFF value of posterior insula (pIC) and non-judgment score (*r* = 0.509, *p* = 0.044) **(C)** of FFMQ.

Within the MBSR group, the mean ALFF value of ROIs located in right anterior cingulate cortex and aIC.L cortex was negatively correlated with the total score (*r* = −0.513, *p* = 0.042) (Figure 3A) and observing score (*r* = −0.520, *p* = 0.0392) (Figure 3B) of FFMQ, respectively. The activation of the posterior insula (pIC) ROI was positively correlated with the non-judgment score (*r* = 0.509, *p* = 0.044) (Figure 3C). A coupling between pIC.L and aIC.L was positively correlated with the non-judgment score (*r* = 0.574, *p* = 0.020). The PANAS negative affect score was positively correlated with ACG.R (*r* = 0.542, *p* = 0.030).

## Discussion

In the present work, as a reliable data-driven approach, ALFF was adopted to map the resting-state functional topology based on the magnitude of spontaneous neural activity. There were two main findings in the current study. Firstly, decreased ALFF was detected in three clusters, including the ACC.R, aIC.L, and pIC.L accompanied by strengthened FC between pIC.L and bilateral amygdala. Both ACC and aIC belong to the SN. While postcentral gyrus belonging to the somatosensory network was observed with increased ALFF. Furthermore, the mean ALFF value of ROIs located in SN showed a correlation with mindfulness scores. Secondly, the core region of the default mode network (DMN), i.e., SFGmed.L, also showed decreased ALFF and a weakened correlation with SN. The postCG.R as another region related to attention control showed an adverse change in activation. Together with a decreased negative affect score, strengthened coupling within SN was also observed in an ROI level analysis. These findings may be of significance in explaining the underlying brain mechanisms for short-time mindfulness practice.

First and foremost, the structural and functional brain changes induced by the 8-week MBSR were similar to traditional long-term meditation practice (Gotink et al., 2016). Among those regions, the cingulate cortex, insula, and dlPFC were major neural underpinnings of mindfulness-based intervention (Bilevicius et al., 2016). In our results, decreased ALFF was observed in the core regions of SN, i.e., ACC and the anterior insula in mindfulness beginners. SN was relevant to the experience and training of mindfulness, given its role in both interoception and redirecting attentional resources (Mooneyham et al., 2016). The first result gives an indication of the outcome of mindfulness practice in relation to attention control.

As mentioned earlier, mindfulness ability was cultivated when interoceptive information was elaborately processed (Paulus et al., 2013). Interoception included receiving, processing, and integrating body-relevant signals together with external stimuli and finally affect motivated behavior (Craig, 2002). It refers to the process of how the brain senses and integrates signals originating from inside the body, providing a moment-by-moment mapping of the body’s internal landscape. This is closely related to one’s state of well-being, energy, and stress levels, as well as mood and disposition (Craig, 2002). Individuals often habitually take the form of escape from the present moment to feel better or to avoid feeling worse. Sensory awareness is achieved to provide a way, which makes that the interoceptive information could be read and then translated into facilitating self-awareness and self-care (Cjp et al., 2019). In this procedure, active attention to the inner body is required. Besides, it has also been told that the integration of interoceptive signals constrains the scope through which cognitive appraisals of well-being occur (Zanna and Cooper, 1974; Farb et al., 2015). Thus, mindfulness and well-being may be bridged basically by interoception. In the framework of mindfulness, focusing on one’s own feelings with acceptance may lead to well-being without expending effort to control negative experiences (Wenzel et al., 2020).

The underlying mechanism could be summarized as bottom-up processing of information. Accumulating evidence from neuroimaging studies mostly support this idea (Westbrook et al., 2013). It was reported that a functional change in insula was induced by MBSR (Sevinc et al., 2018) or MBSR tasks (Farb et al., 2013), as well as dispositional mindfulness (Laneri et al., 2017). Both anterior and posterior parts of the insula were normally discussed. The anterior insula could be activated both in interoceptive awareness (Cui et al., 2020) and emotion perception task (Lamm et al., 2010) including pain perception (Farb et al., 2013). It has been proposed that an emotional change is always accompanied by a physiological change. Being consciously aware of bodily signals could lead to an understanding and acceptance of the feeling states of one’s own body (Kuehn et al., 2015). Therefore, emotional improvement could be a major effect of body-awareness enhancement. It has been reported in detail that mindfulness could promote the precision of afferent signals (Paulus et al., 2013; Duquette, 2017) as the result of increased sensory attention, then increase perceptual inference processing of prediction errors in support of learning that can lead to the adjustment of behaviors (Duquette, 2017). That being the case, it is reasonable to assume that mindfulness practice could increase the efficiency in processing of interoceptive information in practitioners. With the promotion of awareness, one may respond rather than react to an internal or external situation (Meppelink et al., 2016). It happens to be the goal of psychotherapy practice in any theoretical model, described of decreased reactive responses and increased moment-to-moment awareness (Duquette, 2017).

Interoceptive awareness probably originates from the anterior insula (Craig, 2009b). As a method to direct intervention/practice, mindfulness was observed to show a regulated effect on anterior insula activity directly Nevertheless, the processing of interoceptive information was dependent on the cooperation of the subregions of both pIC and aIC. Those two regions were assumed to supply objective and subjective representations of the physical conditions, respectively (Craig et al., 2000; Kuehn et al., 2015; Meppelink et al., 2016). The signal flows through the posterior-to-anterior axis making it possible for the integration of objective interoceptive information. However, the processing could be modified by focusing on bodily sensations. A previous study showed that in a state of bodily focusing, pIC inhibition could decrease the processing of other interoceptive (Kuehn et al., 2015; Meppelink et al., 2016). Moreover, low baseline activity coupled with a high response in the anterior insula was generally observed in experienced meditators (Lutz et al., 2013). Experienced mindfulness meditators are able to attenuate reward prediction signals in a passive conditioning task, which may be related to interoceptive processes encoded in the pIC (Kirk and Montague, 2015). In the current work, the decreased activity of both pIC and aIC in the resting state happens to repeat the results in experienced meditators. Moreover, to a certain extent, both regions showed a correlation with mindfulness ability (especially non-judgment and acceptance facet) to a certain extent. This may indicate that insula is involved in the processing of interoceptive information as a way of acceptance in mindfulness beginners.

Anterior cingulate cortex was related to the computation of prediction errors based on one’s current state and the expected state (Paulus et al., 2013), which resulted in the motivation of individuals for approaching or avoiding stimuli (Holroyd and Yeung, 2012) to restore balance (Khalsa and Lapidus, 2016). Similarly, mindfulness-related structural and activation changes in ACC were also captured by MRI studies. Individuals who were more mindful of the present had greater gray matter volume in the ACC (Lu et al., 2014). The activation of ACC was observed in breath-focused mindfulness tasks (Lazar et al., 2000; Hölzel et al., 2007), deep meditation (Craigmyle, 2013), and decreased in experienced meditators when converting to a state of mindfulness (Ritskes et al., 2003). Mindfulness effects were detected with the decoupling of resting-state FC (rs-FC) between subgenual ACC and amygdala (Taren et al., 2015) or other craving-related regions (caudate, ventral striatum, premotor cortex, and insula) (Westbrook et al., 2013). Both insula (especially the anterior part) (Craig, 2009a) and ACC (Hakamata et al., 2013) were reported to be of great importance in mindful awareness. Attenuation of the right anterior insula and ACC was related to the loaded breathing task (Haase et al., 2014). As to the connection within SN, greater SN connectivity in pIC (but not aIC) and intrinsic connectivity of all insular functional subdivisions to SN regions (including the anterior insula, orbitofrontal cortex, ventral striatum, and midbrain) correlated with a greater interoceptive accuracy (Chong et al., 2017). In the current study, alleviated activation of ACC in the resting state also duplicated that decreased pattern in experienced meditators (Ritskes et al., 2003). Besides, the negative affect score was positively correlated with the activation of ACC, which may indicate the role of this region in mindfulness-related emotional improvement.

Except for the changes mentioned above, the activation of ACC and insula was detected by neuroimaging studies in several self-related processing, such as self-criticism and self-reassurance (Longe et al., 2010). Both ACC and insula were crucial structures in self-evaluation and satisfaction. In the current study, decreased spontaneous activity in the resting state was observed in the right ACC and left insula (including both anterior and posterior parts). This was quite similar to the increased activation observed in depression (Orenius et al., 2017) and decreased skilled practitioners. It has been proposed that the attenuated self-related process was related to emotional amelioration either (Verplanken et al., 2007). Furthermore, in the second part of the current results, SFGmed belongs to the midline structure that is also associated with the self-related process (Orenius et al., 2017; Terpou et al., 2019). The decrease of this region also makes it possible for individuals to achieve a state of mind for the reduction of negative thoughts. Similar to previous reports with the same sample, the regions of DMN were detected with a functional change after MBSR. In the current study, the direct investigation was used rather than an ROI analysis in a previous study (Xiao et al., 2019). However, except for the consideration of methodology, mindfulness under the scope of DMN still needs to be implemented in future. “The mediation effects of DMN on mindfulness and behavioral performance outcomes as well as DMN and its role in individual behavior performance” were nominated (Nien et al., 2020).

As to the divergence across studies, various impact factors can be distilled to the heterogeneity. For instance, the subregions of cingulate cortex have been reported with functional and structural changes related to mindfulness and meditation. Among these studies, long-term, short-term, and trait mindfulness (Lu et al., 2014) with different study designs, sample sizes, demographics, meditation styles, and cingulate subdivisions were used as a reference (Zsadanyi et al., 2021). As proof, the subgenual cingulate cortex was observed with lower rates of annual tissue loss in long-term meditators, suggesting a protective effect of meditation on the emotional and cognitive function (Kurth et al., 2021). The protective effects have also been indicated by short-term body-mind training (Tang et al., 2020). While, in the current study, right ACC was revealed to have alleviated spontaneous activity in post-MBSR assessment and negatively related to mindfulness ability. The divergent results may be attributed to demographic factors, meditation styles, and experience, as well as practice terms. In our previous research work, left medial cingulate cortex functional change was detected (Xiao et al., 2019). The neuroimaging index adopted may explain the variation of the results. For details, ALFF in the current study was considered to be a reflection of neuroactivities in focal regions (Zang et al., 2007), while ReHo adopted previously may characterize the cohesiveness in neighboring brain regions (i.e., focal connectivity) (Zang et al., 2004). Recent works on clinical population have also imposed the focusing on neural correlates of somatic and attention mechanisms underlying the emotional process (Hatchard et al., 2020). Future studies focusing on clinical phenotypes may provide more specific indicators for detecting the interactive process.

Though it may offer some profound and important conclusions, in this study, there are also some limitations that must be taken into consideration. First, there is a moderately small sample size in the current work, which limited the detection of small differences and inflated our chances of revealing positive findings. Secondly, the design of current study only reflects the mindfulness practice as a whole rather than the effects of its component. This could be further studied by using different interventions. Though all mindfulness practices could increase the positivity of effect, energy, and present focus and decreased thought distraction, each of them still presents distinct psychological fingerprints. Besides, a self-reported study also revealed that a positive effect and a reduction of a negative effect would be elicited by being attentive and accepting of unpleasant experiences, respectively (Blanke et al., 2017). In the current study, a typical MBSR procedure was adopted, which seemed to be a compound of the abovementioned practices. It was absolutely necessary to verify which part is more critical in promoting well-being. By answering this, a future study may help to enhance the different aspects of effective well-being by addressing specific facets of mindfulness.

To sum up, after 8-week MBSR training, namely a decrease in the activity of the SN was detected. Besides, enhanced rs-FC was detected in the coupling with or within SN. The correlation of corresponding nodes was related to mindfulness ability and emotional assessment. Those results may shed light on the effect of mindfulness on the brain mechanisms of novice practitioners.

Though no direct relation was detected between emotion and mindfulness ability, our results still showed a decreased negative effect induced by short-term mindfulness practice. The brain regions related to attention and interoception can be involved in the underlying mechanism. Further exploration of the relationship between these brain regions and the corresponding body sensory after mindfulness training is needed. Both subjective and objective evaluations of the internal perceptual awareness or attention should be involved. In short, those results suggested that emotional regulation can be achieved after the beginning of an 8-week mindfulness exercise in healthy volunteers. The accompanying decrease of activity in the SN can provide new clues in elucidating the brain mechanisms of mindfulness novices.

## Data Availability Statement

The raw data supporting the conclusions of this article will be made available by the authors, without undue reservation.

## Ethics Statement

The studies involving human participants were reviewed and approved by Medical Ethics Committee, Kunming University of Science and Technology. The patients/participants provided their written informed consent to participate in this study.

## Author Contributions

QG and ND contributed to data collection, data processing, data analysis, statistical analysis, original manuscript drafting, and manuscript editing. GB, RL, and XZ contributed to data collection, data processing, and data analysis. JZ, SW, and YZ contributed to project conception and manuscript revision. YF and LC helped to perform the analysis with constructive discussions and revised the manuscript. ZC and KW contributed to project conception, research design, and manuscript revision. All authors contributed to the article and approved the submitted version.

## Conflict of Interest

The authors declare that the research was conducted in the absence of any commercial or financial relationships that could be construed as a potential conflict of interest.

## Publisher’s Note

All claims expressed in this article are solely those of the authors and do not necessarily represent those of their affiliated organizations, or those of the publisher, the editors and the reviewers. Any product that may be evaluated in this article, or claim that may be made by its manufacturer, is not guaranteed or endorsed by the publisher.

## Footnotes

1
http://www.restfmri.net

2
http://www.fil.ion.ucl.ac.uk/spm/software/spm8
